# The validation, reliability, and measurement invariance of the cognitive fusion questionnaire in Chinese community-dwelling adults

**DOI:** 10.1186/s40359-025-03011-5

**Published:** 2025-07-01

**Authors:** Harry Ka-shing Chung, Meiqi Xin, Qian Li, Jiaer Lin, Xue Yang, Peter Ka-chun Ho

**Affiliations:** 1 New Life Psychiatric Rehabilitation Association, 332 Nam Cheong Street, Kowloon, Hong Kong SAR China; 2 Department of Rehabilitation Sciences, The Hong Kong Polytechnic University, Kowloon, Hong Kong SAR China; 3 Mental Health Research Centre, The Hong Kong Polytechnic University, Kowloon, Hong Kong SAR China; 4 5/F, Centre for Health Behaviours Research, The Jockey Club School of Public Health and Primary Care, The Chinese University of Hong Kong, Sha Tin, Hong Kong SAR China

**Keywords:** Cognitive fusion, Psychometric properties, Measurement invariance, Chinese, Community-dwelling adults

## Abstract

**Background:**

There was no study on the validation and measurement invariance test of the Cognitive Fusion Questionnaire (CFQ) among Chinese community-dwelling adults. This study aimed to evaluate the reliability, validity and measurement invariance of the 7-item CFQ for cognitive fusion among Chinese community-dwelling adults in Hong Kong.

**Methods:**

In total, 552 Chinese adults (18–64 years old; 51% males) in Hong Kong finished the online survey during March to April 2023. The questionnaire included the CFQ, the Acceptance and Action Questionnaire-II (psychological flexibility), the Kessler Psychological Distress Scale (psychological distress), the Short Warwick-Edinburgh Mental Wellbeing Scale (mental well-being), and the self-rated mental health. Confirmatory factor analysis (CFA) and multi-group CFA were conducted.

**Results:**

The unidimensional structure of the scale was confirmed by CFA, with a satisfactory fit to the sample (χ^2^(14)= 15.935, *p* = 0.317, CFI = 0.998, RMSEA = 0.024, SRMR = 0.021). The standardized factor loadings ranged from 0.71 to 0.78. Full scalar invariance across educational level and psychological distress level and partial scalar invariance across gender and age groups were confirmed by multi-group CFA. The CFQ showed good internal consistency (Cronbach’s α = 0.90), and convergent validity with significant correlations with experiential avoidance (*r*_*s*_(430) = 0.69, 95% CI 0.63 to 0.74), psychological distress (*r*_*s*_(430) = 0.61, 95% CI 0.55 to 0.67), mental wellbeing (*r*_*s*_(430) = -0.29, 95% CI -0.38 to -0.20) and self-rated mental health (*r*_*s*_(430) = -0.24, 95% CI -0.33 to -0.15), with *p*-values all < 0.001.

**Conclusions:**

This study is the first to examine the psychometric properties of the 7-item CFQ in Chinese community-dwelling adults in Hong Kong. The CFQ is a brief, reliable and valid measure for assessing CF among Chinese community-dwelling adults. Caution is warranted for direct comparison across samples with uneven age and gender distributions.

**Supplementary Information:**

The online version contains supplementary material available at 10.1186/s40359-025-03011-5.

## Introduction

Cognitive fusion (CF) is defined as “the tendency for behavior to be overly regulated and influenced by cognition” [1]. Individuals with higher levels of CF treat their thoughts as facts, resulting in cognitive events dominating their behavior and subjective experience, while reducing sensitivity to immediate consequences [1]. Conversely, cognitive defusion can enhance behavioral effectiveness by prioritizing meaningful behaviors and minimizing the impact of distressing thoughts [2]. CF has shown positive relationships with a wide range of psychiatric disorders (e.g., eating disorders [3], anxiety and depression [4–6]) and psychological properties (e.g., psychological distress [4], well-being [7] and experiential avoidance [4]). The underlying psychological mechanisms may involve individuals with CF automatically assigning literal interpretations to negative cognitive events, viewing negative thoughts and distorted self-perceptions as factual, thereby intensifying the experience of negative emotions and inflexible responses [8].

Mental health disorders have been a significant public health concern in China in recent years, with 5.5% of the general population exhibiting a high depression risk (measured by the 9-item Patient Health Questionnaire Depression Scale with a cutoff of 10) and 2.8% of a high risk of anxiety (measured by the 7-item Generalized Anxiety Disorder with a cutoff of 10) in 2024 [9]. This evidence underscores the urgent need for early identification and interventions to reduce the burden of mental illness and prevent severe outcomes such as suicide [10]. As a robust predictor of mental health disorders, CF has received growing attention in both research and clinical practice [11]. It has been a key concept and has been widely evaluated in psychological interventions for mental health, such as Acceptance and Commitment Therapy (ACT) [12], Mindfulness-based Cognitive Therapy [13], and Cognitive Behavioural Therapy (CBT) [14]. Hence, a validated and culturally adapted measurement tool for CF is needed, which can facilitate early identification of various mental disorders and enhance the understanding of the underlying mechanisms of these interventions.

Several measures have been used to measure CF but only in a specific context of a mental problem, such as the Believability of Anxious Feelings and Thoughts Questionnaire (BAFT) (for anxiety) [15], the Drexel Defusion Scale (DDS) (for social anxiety) [16], and the Automatic Thoughts Questionnaire (ATQ) (for depression) [17]. The Avoidance and Fusion Questionnaire (AFQ) [18, 19] assesses multiple processes of ACT, including avoidance and fusion. In contrast, the short-form single-dimension Cognitive Fusion Questionnaire (CFQ), including 7 items, is a well-developed measure of the general concept of CF that can be used for diverse populations and contexts [1]. The initial 44-item CFQ [1] was constructed and validated among adults in the UK, which covered a broad functional definition of fusion, including believability of thoughts, taking thoughts literally, trying to control thoughts, overanalyzing situations, evaluating thought content, dominance of cognition in a person’s experience, perspective taking, and detached awareness of thoughts. The refined 7-item version retains strong psychometric properties while enhancing practical applicability [1].

The English version of the 7-item CFQ has demonstrated strong reliability (Cronbach’s α = 0.88 to 0.93), validity (significant positive correlations with the scales measuring psychological flexibility/experiential avoidance), and discriminant validity across diverse populations, including students, community members, working individuals, and clinical samples [1]. It has also been successfully validated in multiple languages and cultural contexts, including Colombia [20], Korea [21], Germany [22], and France [23]. However, to date, the CFQ has not been validated among Chinese community-dwelling adults. We identified three studies published in Chinese journals: Two of them evaluated the 13-item and 9-item CFQ in college students [24] and breast cancer patients [25], respectively. One study assessed the 7-item CFQ in ethnicts minority cenollege [27] stud[26]. These studies recruited either college students or patients, which restricts the generalizability of the findings to the general population. To our knowledge, no validation studies of the CFQ have been conducted among Chinese community-dwelling adults, particularly in Hong Kong, where people speak Cantonese. Furthermore, no study has examined the measurement invariance of the CFQ across key demographic variables such as gender, age and education level. Given that psychometric properties may vary across cultural and demographic groups [28–30], it is necessary to test the validity and measurement invariance of the CFQ in a broader Chinese community population.

This study aimed (1) to validate the 7-item CFQ in the Chinese community-dwelling adult population in Hong Kong to establish its psychometric properties by analyzing its dimensionality, reliability, and convergent validity; and (2) to test the measurement invariance across gender, age, education level and distress level. It is hypothesized that (1) the total CFQ scores would be positively associated with psychological distress, experiential avoidance and negatively associated with mental well-being; (2) measurement invariance would be established across different groups of gender, age, education level and distress level.

## Method

### Participants

The present cross-sectional study involved a sample of 552 Chinese community-dwelling adults living in Hong Kong. Participants were recruited from the social media platform of one of the largest mental health agencies in Hong Kong and through email invitations to the networks of a local university (Note: the agency name is omitted for blind peer review) during March to April 2023. Inclusion criteria included: (1) being aged 18 or above, (2) having the ability to read and understand Chinese, and (3) having access to the internet for the online questionnaire. Individuals who were unable to independently complete the entire questionnaire or did not provide informed consent were excluded from the study. The online questionnaire was implemented on Qualtrics, a widely used online survey platform, via an invitation link. All participants provided informed consent after reviewing the study terms and instructions. On average, participants took approximately 18 min to complete the questionnaire anonymously, unless they opted to receive a voucher worth HKD$ 20 (USD$ 2.5) as a token of their participation. Ethical approval was obtained from the ethics committee of the PolyU Institutional Review Board (Ref.# HSEARS20221222002).

### Measures

#### Cognitive fusion questionnaire (CFQ)

To assess cognitive fusion within the Hong Kong population, a thorough review procedure was conducted on the 9-item Simplified Chinese version of the Cognitive Fusion Questionnaire (CFQ) of Wang et al. [26, 27]. First, an experienced bilingual research assistant with a background in psychology translated the items from Mandarin to Traditional Chinese. Second, authors HKC (an eligible psychologist), KCH (a social worker supervisor), and MX (a scholar in cross-cultural research) reviewed the items and ensured the cultural appropriateness and accuracy of the translated wordings. Given that the original English version of the CFQ demonstrated that a 7-item version was sufficient for obtaining reliable and valid results [1], the current analysis excluded Q6 (“I need to control the thoughts that come into my head”) and Q8 (“I tend to react very strongly to my thoughts”) in the Madarin version. Participants rated the items using a 7-point Likert scale, ranging from 1 (“never true”) to 7 (“always true”). A higher total score on the CFQ indicates a stronger level of cognitive fusion. The Traditional Chinese version of CFQ is shown in Appendix 1.

#### Acceptance and action questionnaire-II (AAQ-II)

The 7-item Chinese version of the AAQ-II was utilized to measure psychological flexibility/experiential avoidance [31]. Participants rated their responses on a 7-point Likert scale, with higher total scores indicating higher psychological inflexibility or greater experiential avoidance. The AAQ-II demonstrated good internal consistency in the present study sample (Cronbach’s α = 0.94).

#### Kessler psychological distress scale (K10)

The Chinese version of the K10 was used to assess psychological distress [32]. It consists of 10 items rated on a 5-point Likert scale, with a total score ranging from 10 to 50. The scores on the K10 are commonly interpreted with the following cut-offs: 10–19 indicating likely well-being, 20–24 indicating likely mild disorder, 25–29 indicating likely moderate disorder, and 30–50 indicating likely severe disorder. The K10 demonstrated good internal consistency in the present study sample (Cronbach’s α = 0.93).

#### Short Warwick-Edinburgh mental wellbeing scale (SWEMWBS)

The short form of the Chinese Warwick-Edinburgh Mental Wellbeing Scale [33] was used to measure mental wellbeing. It utilizes a 5-point Likert scale, with higher total scores indicating better mental wellbeing. The SWEMWBS demonstrated satisfactory internal consistency in the present study sample (Cronbach’s α = 0.84).

#### Self-rated mental health (SRMH)

The single-item measure of self-rated mental health asks respondents to rate their mental health on a five-point scale ranging from excellent to poor [34]. The score is reverse-coded, meaning that a higher score indicates better self-rated mental health. SRMH is increasingly used in health research and population health surveys [34].

### Data analysis

A single-group confirmatory factor analysis (CFA) was conducted to evaluate the hypothesized single-factor structure of the CFQ. Multi-group CFA was performed to assess the measurement invariance of the CFQ across different demographic backgrounds (male versus female; aged 34 or below [young-aged group] versus aged 35 or above [middle-aged group]; diploma or below versus degree or above) and levels of distress (null/mild psychological distress versus moderate/severe psychological distress). The measurement invariance testing procedures outlined by Fischer & Karl [35] were followed, and a hierarchical approach was used to constrain a number of parameters across the groups. A test for multivariate normality and both the skewness and kurtosis of the seven CFQ items indicated that the seven items did not show a multivariate normal distribution. Therefore, the Maximum Likelihood (MLM) estimator was employed for model estimation, in which robust standard errors and chi-square test statistics were adjusted for non-normality.

Reasonable goodness of fit was indicated by the comparative fit index (CFI > 0.90), the root mean square error of approximation (RMSEA < 0.08), and the standardized root mean square residual (SRMR < 0.08) [36]. The null hypothesis of measurement invariance was rejected when there was a change in CFI (∆CFI) exceeding 0.01, along with a change in RMSEA (∆RMSEA) above 0.015, or a change larger than 0.03 in SRMR (∆SRMR). To establish partial measurement invariance, non-invariant individual items were identified using modification indices (MIs).

Convergent validity was examined by assessing the Spearman’s rank-order correlation between the CFQ and other well-validated measures, including AAQ-II, K10, SWEMWBS, and SRMH.

All analyses were conducted using R (2022.07.0 Build 548). Two-tailed *p*-value < 0.05 was considered statistically significant.

## Results

### Data validation and descriptive statistics

The following data validation procedures were applied to the collected sample. Participants were excluded from analysis if they: (1) completed the survey with an unusually short duration (i.e., 300 s); (2) completed the survey more than once, as evidenced by duplicate records of name and telephone number; or (3) provided a CFQ score of 3 standard deviations or higher. Consequently, a final sample comprising 432 observations was formed. Given that CFQ is hypothesized to be a single factor model with 7 indicators, this sample size for CFA and multi-group CFQ was deemed sufficient based on previous research [37]).

Table 1 presents an overview of the demographic and clinical characteristics of the valid sample. There were 220 males (50.9%) and 212 females (49.1%). Around 80% of the participants were distributed in age groups between 25 and 44 years old, and more than one-third of them had severe psychological distress according to K10.

**Table 1:** Characterization of the samples (*n* = 432)

	Frequency (% of the sample)	Mean scores of CFQ (SD)
Demographics:		
Age:		
18–24 25–34 35–44 45–54 55–64	44 (10.2) 249 (57.6) 109 (25.2) 26 (6.0) 4 (0.9)	29.98 (9.94) 33.63 (6.63) 30.57 (8.38) 34.15 (8.08) 26 (11.66)
Sex:		
Male	220 (50.9)	31.99 (8.02)
Female	212 (49.1)	32.92 (7.49)
Educational level:		
Diploma or below	183 (42.4)	33.67 (6.44)
Degree or above	249 (57.6)	31.55 (8.51)
Distress level:		
Psychological well	123 (28.5)	25.41 (8.03)
Mild	74 (17.1)	32.59 (6.29)
Moderate	73 (16.9)	34.41 (4.67)
Severe	162 (37.5)	36.83 (5.10)

### Scale structure

**Fig. 1:**
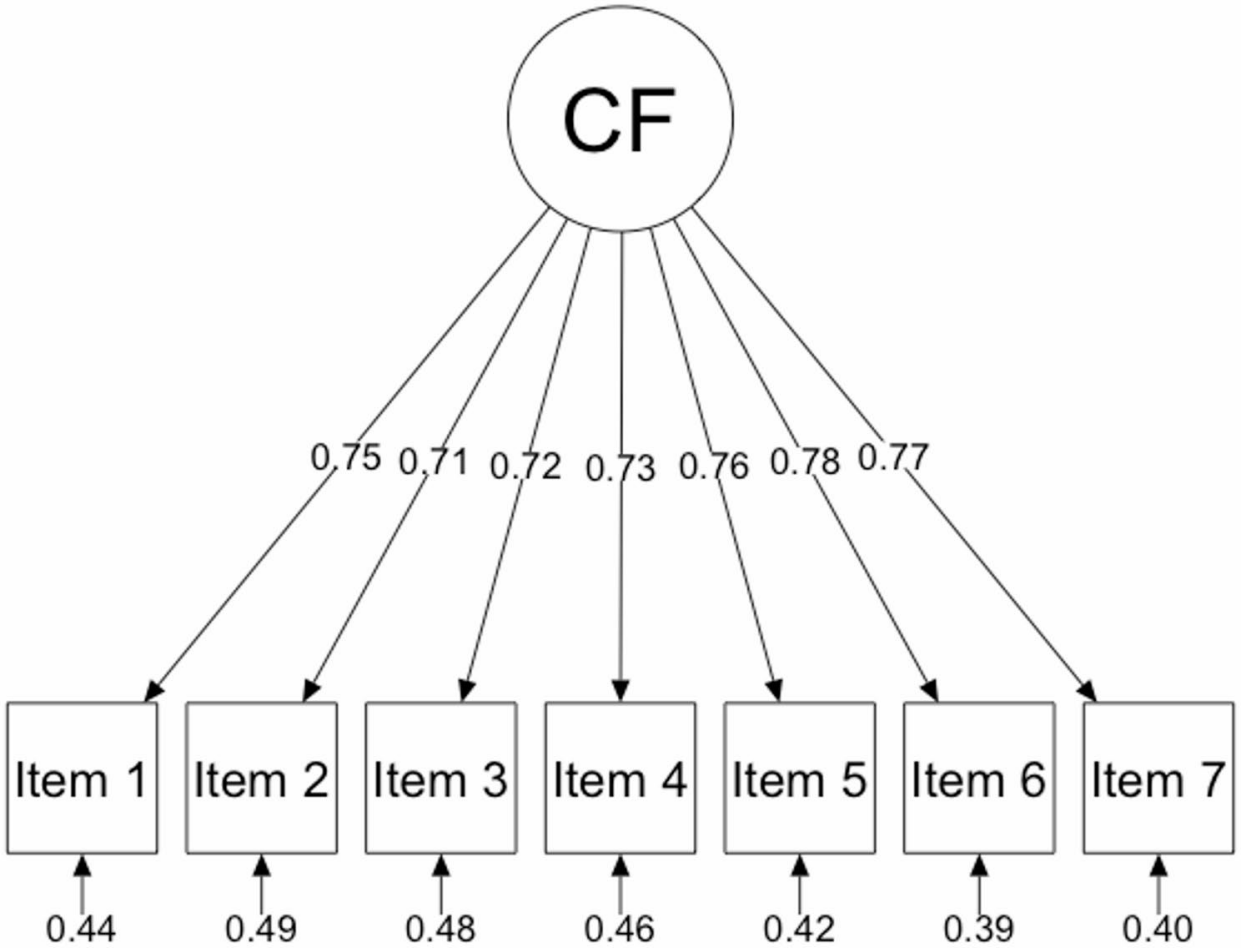
Path diagram for single-group confirmatory factor analysis of cognitive fusion questionnaire. Note: Standardized estimates are displayed. CF = Cognitive Fusion

The results of the single-group CFA supported the single-factor model of the 7 CFQ items, as evidenced by a satisfactory fit to the sample (scaled χ^2^(14)= 15.935, *p* = 0.317, CFI = 0.998, RMSEA = 0.024, SRMR = 0.021). The standardized factor loadings ranged from 0.71 to 0.78 as shown in Fig. 1.

### Measurement invariance

Measurement invariance was tested using multi-group CFA within a structural equation modeling framework. For each grouping variable (gender, age, education, distress level), invariance was assessed sequentially across three hierarchical levels. First, configural invariance was established by confirming that the factor structure (i.e., number of factors and pattern of item-factor loadings) was equivalent across groups, with no equality constraints. Second, the metric invariance model imposed equality constraints on factor loadings across groups, and the change in model fit was compared to that of the configural model. Finally, the scalar invariance model imposed equality constraints on both factor loadings and item intercepts across groups, with model fit compared to that of the metric model. If full scalar invariance was unsupported, partial scalar invariance models were estimated by selectively freeing non-invariant intercepts, which were identified via modification indices. Table 2 provides a summary of the measurement invariance results. The configural invariance and metric invariance models were demonstrated across gender, age, education, and psychological distress level. However, full scalar invariance was demonstrated in the groups of education and psychological distress level, but not in the groups of gender and age. Examination of modification indices suggested that the intercepts of items 1 and 3 should be allowed to vary across gender and age, respectively. Partial invariance models were supported in the modified models for gender and age.

**Table 2:** An overview of the measurement invariance results

Model	χ^2^	df	CFI	RMSEA	SRMR	∆χ^2^	∆df	∆CFI	∆RMSEA	∆SRMR
	Gender (female: *n* = 212; male: *n* = 220)									
Configural	53.409	28	0.995	0.038	0.026	NA	NA	NA	NA	NA
Metric	60.559	34	0.993	0.038	0.041	+ 8.659	6	-0.002	+ 0.000	+ 0.015
Scalar	90.709	40	0.977	0.065	0.051	+ 30.429***	6	-0.016	+ 0.027	+ 0.010
-Partial	69.065	39	0.991	0.042	0.044	+ 8.569	5	-0.002	-0.004	+ 0.003
	Age (aged 34 or below: *n* = 293; aged 35 or above: *n* = 139)									
Configural	52.370	28	0.998	0.024	0.026	NA	NA	NA	NA	NA
Metric	54.878	34	0.999	0.014	0.033	+ 3.298	6	+ 0.001	-0.010	+ 0.007
Scalar	75.166	40	0.989	0.044	0.040	+ 20.303**	6	-0.010	+ 0.030	+ 0.007
-Partial	64.967	39	0.995	0.029	0.036	+ 10.055	5	-0.004	+ 0.015	+ 0.003
	Education (diploma or below: *n* = 183; degree or above: *n* = 249)									
Configural	94.425	28	0.970	0.088	0.038	NA	NA	NA	NA	NA
Metric	103.308	34	0.968	0.083	0.050	+ 11.348	6	-0.002	-0.005	+ 0.012
Scalar	109.507	40	0.967	0.077	0.052	+ 6.141	6	-0.001	-0.006	+ 0.002
	Psychological distress level (null/mild distress: *n* = 197; moderate/severe distress: *n* = 235)									
Configural	64.919	28	0.984	0.056	0.034	NA	NA	NA	NA	NA
Metric	76.156	34	0.979	0.057	0.051	+ 11.872	6	-0.005	+ 0.001	+ 0.017
Scalar	93.708	40	0.969	0.064	0.062	+ 16.282*	6	-0.010	+ 0.007	+ 0.011

Note. χ^2^, Standard Chi-square; df, Degree of freedom; CFI, Robust Comparative Fit Index; RMSEA, Robust Root Mean Square Error of Approximation; SRMR, Standardized Root Mean Square Residual; ∆, change in value; *p < 0.05, **p < 0.01, ***p < 0.001

### Reliability and convergent validity

Table 3 displays the item statistics of the CFQ. Good internal consistency was demonstrated for the scale (Cronbach’s α = 0.90). To establish convergent validity, correlational analyses were conducted between the CFQ and other variables. The results indicated that the CFQ had strong positive associations with experiential avoidance, as measured by AAQ-II (*r*_*s*_(430) = 0.69, 95% CI 0.63 to 0.74, *p* < 0.001), and psychological distress, as measured by K10 (*r*_*s*_(430) = 0.61, 95% CI 0.55 to 0.67, *p* < 0.001). In contrast, weak negative associations were observed between the CFQ and mental wellbeing, as measured by SWEMWBS (*r*_*s*_(430) = -0.29, 95% CI -0.38 to -0.20, *p* < 0.001) and self-rated mental health (*r*_*s*_(430) = -0.24, 95% CI -0.33 to -0.15, *p* < 0.001).

**Table 3:** Summary of item statistics

	Mean (SD)	Skewness	Kurtosis	Corrected item-whole correlation
Item 1	4.67 (1.48)	-0.50	2.79	0.74
Item 2	4.51 (1.40)	-0.71	2.86	0.71
Item 3	4.52 (1.42)	-0.62	3.03	0.72
Item 4	4.63 (1.46)	-0.53	2.61	0.73
Item 5	4.56 (1.40)	-0.55	2.92	0.75
Item 6	4.72 (1.33)	-0.43	2.80	0.77
Item 7	4.83 (1.37)	-0.74	3.27	0.77

## Discussion

This study, to our best knowledge, is the first to examine the psychometric properties of the 7-item CFQ in Chinese community-dwelling adults. The findings suggest that the CFQ is a reliable and valid unidimensional self-report measurement for cognitive fusion in this population. Configural invariance and metric invariance were established across gender, age, education, and psychological distress levels. While full scalar invariance held for education and psychological distress levels, partial scalar invariance was demonstrated for gender and age. Besides, the CFQ showed good internal reliability (Cronbach’s α = 0.90) and significant item-whole correlations (all *r* > 0.50). This is consistent with previous validation studies on the CFQ conducted among German (Cronbach’s α = 0.94) [22] and Spanish (Cronbach’s α = 0.96) [2] populations. The structural validity of the CFQ was supported by the CFA model, which showed a good model fit to the data with all standardized factor loadings exceeding 0.39, indicating moderate effect sizes of each item on the concept of cognitive fusion.

Convergent validity was also established, as the CFQ demonstrated strong positive correlations with experiential avoidance and psychological distress. It is consistent with previous research conducted in American adults (*r* ranged from 0.57 to 0.76) [4]. Weak negative correlations were found between the CFQ and mental well-being and self-rated mental health in our study. In contrast, a study conducted among American undergraduates showed a strong negative correlation (*r* = -0.68) between the CFQ and mental well-being [38]. This notable difference in correlation strength may reflect socio-cultural differences in how cognitive fusion relates to well-being between the two constructs. Future studies should explore whether cognitive fusion impacts well-being through culture-specific pathways.

One of the main contributions of this study is its first attempt to assess the measurement invariance of the CFQ across demographic characteristics, which indicates whether its latent construct is measured equivalently across different groups [39]. Measurement invariance of the 7-item CFQ was generally established across demographic characteristics and psychological distress level. Specifically, the results showed the CFQ exhibited configural and metric invariance across age, gender, education level, and distress level, which indicated that the CFQ could be used across these groups with meaningful interpretation of measurement data [39]. Similarly, the metric invariance of CFQ across gender was reported among Colombian adults [20]. However, full scalar invariance was not supported for age and gender in our study, and items 1 (“My thoughts cause me distress or emotional pain”) and 3 (“I over-analyze situations to the point where it’s unhelpful to me”) were identified to contribute to the variance. This suggests that the mean levels of items 1 and 3 would vary across gender and age, respectively, and direct comparisons of CFQ mean scores between these groups might not be appropriate in the Chinese population. Future studies should conduct qualitative interviews in these groups to better understand how they understand and endorse the two items differently. It is recommended to compare CFQ scores while adjusting for the uneven distribution of age and gender, or to conduct subgroup analyses, particularly for cross-sectional between-subject studies. The finding highlights the importance of testing the measurement invariance of the scale in different groups instead of assuming its invariance across populations [40].

### Limitations

Several limitations of this study should be noted. First, the cross-sectional study design could not test the predictive validity of the CFQ. Future longitudinal research is needed to evaluate this psychometric property. Second, the sample of this study was primarily comprised of young and middle-aged adults aged between 18 and 54 years old. This could be attributed to the convenience sampling approach employed through social media and online invitations. There are some inherent drawbacks associated with utilizing an online sample. For instance, older individuals who are less inclined to actively engage with social media platforms might have been excluded. In addition, this study did not include clinical samples with diagnosed psychological distress or mental health problems, nor did it establish cutoff values for discriminating levels of CF. Future studies on the diagnostic validity of the CFQ in Chinese adults are needed. Third, as the study was conducted exclusively in one Chinese community in Hong Kong, measurement invariance in cultural groups was not tested. Fourth, test-retest reliability was not conducted in the current study. Also, lay participants were not involved in cognitive interviews to assess the face validity of the questionnaire. These issues should be addressed in future validation studies.

## Summary

This study assessed the psychometric properties of the 7-item CFQ among community-dwelling adults in Chinese. Results suggested that CFQ was an appropriate measurement for assessing the CF level, demonstrating good reliability and validity. Full and robust scalar invariance was demonstrated across educational level and psychological distress level, while partial scalar invariance across gender and age groups was identified. It is advisable to interpret the scores of the variant items with caution for direct comparison across samples with uneven age and gender distributions. For researchers, the established configural, metric and partial scalar invariance allows for meaningful comparisons of structural associations and latent means across different demographic groups. This study highlights that cross-group equivalence is not self-evident, and cross-group equivalence as a prerequisite for latent comparisons across different demographic groups remains an important research subject for future research on the CF. For practitioners, the availability of group-specific scores enhances the interpretability of the CF in clinical and educational settings and the understanding of how CF may vary across the lifespan and between genders. Future work should extend validation efforts to other demographic groups, such as sexual minorities and immigrants, to establish broader applicability.

## Electronic supplementary material

Below is the link to the electronic supplementary material.

Supplementary Material 1

## Data Availability

The data that support the findings of this study are available on request from the corresponding author.
