# Supplementary material for: The validation, reliability, and measurement invariance of the cognitive fusion questionnaire in Chinese community-dwelling adults
Source: BMC Psychol. 2025 Jul 1;13:629. doi: 10.1186/s40359-025-03011-5 (PMC12210738; doi:10.1186/s40359-025-03011-5)
Supplement: Supplementary file 1 — Supplementary Material 1 [file 40359_2025_3011_MOESM1_ESM.docx]

|  | Original English version | Translated traditional Chinese version |
| --- | --- | --- |
| Item 1 | My thoughts cause me distress or emotional pain | 某些想法使我感到煩惱和痛苦 |
| Item 2 | I get so caught up in my thoughts that I am unable to do the things that I most want to do | 我被某些想法困擾以致無法完成要做的事情 |
| Item 3 | I over-analyse situations to the point where it’s helpful to me | 我過分地分析某些情況，但這對我毫無用處 |
| Item 4 | I struggle with my thoughts | 我在自己的某些想法中掙扎 |
| Item 5 | I get upset with myself for having certain thoughts | 我為某些想法感到心煩意亂 |
| Item 6 | I tend to get very entangled in my thoughts | 某些想法讓我很糾結 |
| Item 7 | It’s such a struggle to let go of upsetting thoughts even when I know that letting go would be helpful | 雖然明白放下最好，但我仍然糾結於某些煩惱的想法 |

Appendix 1. The Traditional Chinese version of cognitive fusion questionnaire
